# Impulsivity links reward and threat sensitivities to substance use: a functional model

**DOI:** 10.3389/fpsyg.2014.01194

**Published:** 2014-10-27

**Authors:** George B. Richardson, Jonathan M. Freedlander, Elizabeth C. Katz, Chia-Liang Dai, Ching-Chen Chen

**Affiliations:** ^1^School of Human Services, University of CincinnatiCincinnati, OH, USA; ^2^Centers for Medicare and Medicaid ServicesBaltimore, MD, USA; ^3^Department of Psychology, Towson UniversityTowson, MD, USA

**Keywords:** reward sensitivity, threat sensitivity, impulsivity, substance use, college students

## Abstract

This study used structural equations modeling and undergraduate student data to examine the effects of reward and threat sensitivities on substance use, along with the extent to which impulsivity explained these effects. Our results suggest that impulsivity may translate inversely related reward and threat sensitivities into substance use, completely mediate the effect between threat sensitivity and substance use, and partially mediate the effect between reward sensitivity and substance use. Our results also suggest that individuals with a combination of higher levels on both reward and threat sensitivities may be most impulsive and vulnerable to heightened substance use. We discuss implications for research at the interface of personality and substance use and also substance abuse prevention and treatment.

## INTRODUCTION

Substance use disorders (SUDs) are among the most prevalent psychiatric disorders in the United States. According to the Substance Abuse and Mental Health Services Administration (SAMSHA, 2013), an estimated 22.2 million persons aged 12 or older, or 8.5% of the population, met the criteria for substance abuse or dependence during the past year. College students are at special risk for substance-related harms. According to SAMSHA (2013), college students are more likely to identify as current drinkers, binge drinkers, and heavy drinkers than their non-college counterparts. About 60% of full-time college students identify as current drinkers and this prevalence persists among college graduates (SAMSHA, 2013). Twenty one percent of full-time students reported that they smoked cigarettes in the past month, while 22% reported using illicit drugs (SAMSHA, 2013). Research suggests that among youth, substance abuse has resulted in delinquent behavior, impaired cognitive functioning, emotional distress, and accidents which have led to serious medical injury and death (Jacobus et al., 2009; Goldstein, 2011; Pompili et al., 2012). More than 500,000 college students have been unintentionally injured each year while drinking alcohol (Hingson et al., 2005). Finally, alcohol and illicit drug use have been linked to lower college grade point average, suggesting that substance use may compromise academic achievement (University of Minnesota, 2007).

Research suggests that personality traits function as risk factors for substance abuse and also impact treatment outcomes (Staiger et al., 2007). This suggests that basic scientific information bearing on the personality correlates of substance use could be used to enhance substance abuse prevention and treatment. In particular, such information could be used to identify at-risk individuals and enable providers to personalize treatments, thereby improving clinical outcomes (Staiger et al., 2007). In addition, some research suggests that drug rehabilitation has moderate effects on Big Five traits, implying that perhaps personality mediates treatment outcomes and could be targeted to facilitate recovery (Piedmont, 2001). Colleges and universities may find new information in this area particularly useful given their vested interest in protecting student health and well-being.

Although researchers have identified a number of personality correlates of substance use, more work is needed to identify how these traits function along causal pathways to substance use initiation and progression to abuse. In this study, we develop a model for conceptualizing the etiologic function of three personality correlates of substance use – reward sensitivity, threat sensitivity, and impulsivity. According to our model, reward and threat sensitivities each cause greater substance use through their effects on impulsivity. We contend that this framework can help resolve inconsistencies in the literature at the interface of personality and substance use and also build upon recent studies of impulsivity. We use structural equations and undergraduate student data to test our model and discuss implications for research, prevention, and treatment.

### REWARD AND THREAT SENSITIVITIES

Reward sensitivity has been defined as the extent to which the behavioral approach system is engaged by incentive cues, while threat sensitivity has been defined as the extent to which the behavioral withdrawal or inhibition system is engaged by the perception of threat (Carver, 2008). Among personality theorists who hold dimensional views of affect, there is broad agreement that traits such as eagerness and excitement reflect reward sensitivity, while traits such as anxiety and self-consciousness reflect threat sensitivity (Staiger et al., 2007; Carver, 2008; Aluja and Blanch, 2011). These conceptualizations are also consistent with evolutionary perspectives on the emotions, which hold that intense positive emotions such as excitement prepare the body to approach bio-energetic and reproductive resources, while fear and anxiety prepare the body to avoid threats (MacDonald, 1995; Buss, 2009). From an evolutionary perspective, greater reward sensitivity implies heightened desire for discovery of resources that can be acquired and consumed (i.e., resource seeking), while greater threat sensitivity corresponds to heightened desire for the experience of harm avoidance.

### REWARD SENSITIVITY, THREAT SENSITIVITY, AND IMPULSIVITY

Impulsivity has been defined as “the tendency to act on cravings and urges rather than reining them in and delaying gratification” (e.g., Lord, 2007), or as a disposition toward rash action in response to strong emotion (e.g., Whiteside and Lynam, 2001; Cyders and Smith, 2007). Some studies have treated impulsivity as a response to facets of reward sensitivity such as excitement, sensation seeking, or latent liability for both sensation seeking and impulsivity (e.g., Zuckerman, 1996; Ames et al., 2002). In contrast, other research has examined impulsivity as a response to facets of threat sensitivity (e.g., negative emotionality; Eisenberg et al., 2005; Whiteside et al., 2005). Similar findings have emerged from studies of normal and abnormal personality, where some researchers have modeled impulsivity as a reflective indicator of an externalizing dimension that has subsumed components of reward sensitivity, including excitement seeking and boredom proneness, along with behavioral correlates of reward sensitivity (e.g., antisocial behavior; Krueger et al., 2007). In seeming opposition, other researchers have modeled impulsivity as reflecting an internalizing dimension that subsumes facets of threat sensitivity (e.g., anxiety; Cosi et al., 2011).

Some research has indicated that impulsivity occurs in the context of intense positive *and* negative emotion (Cyders and Smith, 2007; Cyders et al., 2007). Cyders and colleagues have named the combination of positive emotionality and impulsivity *positive urgency*, while the combination of negative emotionality and impulsivity has been termed *negative urgency* (Whiteside et al., 2005). The urgency items appear to combine several affects with impulsivity. For instance, some positive urgency items refer to impulsivity in the context of excitement, while others refer to impulsivity in the context of happiness. While these combinations of item content could make precise causal modeling of the effects between affects and impulsivity difficult, studies of urgency do seem to suggest that manifestations of reward and threat sensitivity may share proximate causes with impulsivity. One possibility is that latent reward and threat sensitivities cause people to behave impulsively and also feel intensely happy or upset. That is, trait intensity of behavioral approach and withdrawal system engagement by incentive and threat cues, respectively, may explain participants’ positive and negative urgency scores.

### REWARD SENSITIVITY, THREAT SENSITIVITY, AND SUBSTANCE USE

A number of studies have linked risky behaviors such as substance use to facets of reward sensitivity (Staiger et al., 2007). For instance, substance use has been tied to the fun seeking and drive facets of behavioral approach (Franken and Muris, 2006), externalizing dimensions (e.g., Krueger et al., 2007), and numerous studies have linked substance use to sensation seeking (e.g., Wood et al., 1995; Arnett, 1996; Ames et al., 2002; Nower et al., 2004; Robbins and Bryan, 2004). In addition, Morgan et al. (2014) found that reward sensitivity predicted alcohol use, along with psychopathic traits and conduct problems.

According to Staiger et al. (2007), an inconsistent picture of the effect between threat sensitivity and substance use has emerged and threat sensitivity may have more to do with the maintenance of SUDs than their etiology. For example, Grau and Ortet (1999) reported that facets of reward sensitivity, but not anxiety-related traits and neuroticism, were significantly correlated with substance use. But in other studies, anxiety-related traits such as harm avoidance have been tied to impulsivity. According to Verheul (2001), harm avoidance may impact substance use indirectly via a pathway through stress responsivity and disinhibition (DIS). Thus, perhaps the effect between threat sensitivity and substance use does not emerge unless threat sensitivity and also impulsivity-type traits are modeled. As another possibility, the effects between facets of threat sensitivity and substance use might have emerged in the Grau and Ortet (1999) study if reward sensitivity had been held constant during their estimation. In other words, threat sensitivity might be a significant predictor of substance use within levels of reward sensitivity.

### IMPULSIVITY AND SUBSTANCE USE

A very large literature has documented that substance use is reliably linked to impulsivity. For instance, Allen et al. (1998) found that drug dependent individuals were more impulsive than those with no history of drug use. Similar Kane et al. (2004) found that women comorbid for eating disorders and alcohol abuse were more impulsive than women with eating disorders only, who in turn were more impulsive than controls. Impulsivity has also been implicated in the initiation of substance use and progression to abuse. In a non-clinical sample of adolescents, Robbins and Bryan (2004) found that impulsivity predicted alcohol problems, alcohol use, condom use, and cigarette smoking. According to Moeller and Dougherty (2002), impulsivity among children and adolescents has predicted higher levels of substance use longitudinally. Moeller and Dougherty (2002) also reviewed evidence that impulsivity has a negative impact on treatment outcomes and is also heightened by drug use. Finally Verdejo-García et al. (2007) reviewed evidence from high-risk population research, problem gambling studies, and genetic association studies that confirm that impulsivity is a vulnerability marker for SUDs.

### A FUNCTIONAL MODEL

Newman and colleagues developed a framework that helps to shed light on the causal relations between reward sensitivity, threat sensitivity, and impulsivity (Wallace et al., 1991; Newman and Wallace, 1993). Newman and colleagues’ framework integrates Eysenck’s personality system with Gray’s neuropsychological model (Gray, 1987; Newman and Wallace, 1993; Whiteside and Lynam, 2001). Gray’s model posits that behavior arises from a behavioral activation system (BAS), a behavioral inhibition system (BIS), and a non-specific arousal system (NAS). The BAS responds to reward or resource-associated environmental stimuli by initiating approach, while the BIS responds to threat-associated environmental stimuli with avoidance or inhibitory behavior. The BAS and BIS are thought to counteract one another. The NAS is activated by the BAS and the BIS and can intensify behavior emanating from either system, promoting rapid responding. We note that this model is consistent with the wide agreement among emotion researchers that valence and arousal dimensions are helpful in describing affective experience (Demaree et al., 2005). Stemming from their research, Newman and Wallace (1993) suggested that whenever individuals experience extensive NAS activation in response to cues for reward or threats, they are likely to respond impulsively, proposing that the NAS is “an adaptive, energizing system that facilitates rapid action in emergency situations” (p. 706).

Newman and colleagues identified three pathways to impulsivity. The first pathway is a reward mediated pathway to increased NAS activity that is associated with Gray’s BAS. The second is a punishment mediated pathway to increased NAS activity that is associated with Gray’s BIS. The final pathway reflects deficits in the integration of BAS and BIS processes and is observed as widespread self-regulatory deficits (Newman and Wallace, 1993). According to Newman and Wallace (1993), the third pathway is underpinned by disintegrated BAS and BIS activity, or in other words, difficulty shifting attention between reward and threats.

Research on the effects of psychoactive substances indicates that the brain experiences many substances as biologically meaningful via their modulation of basic motivational systems (e.g., they impact dopamine transmission and incentive salience), meaning they provide the brain with information signaling the discovery of valuable resources and/or the avoidance of threats (Nesse, 1994, 2002; Lende and Smith, 2002; Goldman et al., 2006; Berridge, 2012). Because substances can provide the experience of resource rich contexts, high levels of reward sensitivity may confer risk for tagging substance-related stimuli as extremely salient or critical resources. Similar, high levels of threat sensitivity may confer risk for identifying substances as an effective means for avoiding critical threats. As the brain prepares the body to respond to stimuli identified as critical resources or threats, individuals have the experience that they must act immediately (i.e., they experience positive or negative urgency; Cyders et al., 2007). Thus, some humans may abuse substances because (1) they are characterized by heightened desire for discovering valuable resources or avoiding threats and (2) substances are experienced as critical resources for achieving success toward these ends (see Richardson and Hardesty, 2012). Given Newman and colleagues’ work and research bearing on the effects of psychoactive substances, we theorize that reward and threat sensitivities each drive substance use through their effects on impulsivity.

### THE CURRENT STUDY

We have developed an integrative and functional model of the effects between reward and threat sensitivities, impulsivity, and substance use. This model may bring some clarity to research at the interface of personality and substance use by providing an account of the function of personality traits and why substance use coordinates with them. For instance, this model may extend upon Cyders and colleagues’ studies of positive and negative urgency by describing how positive affects (e.g., excitement) and impulsivity, which combine to form urgency, are related.

This model may also help to resolve inconsistent findings we discussed in *Reward Sensitivity*, *Threat Sensitivity*, *and Substance Use* regarding the effect(s) between threat sensitivity and substance use. Further, by linking impulsivity to externalizing and also internalizing traits, it might point the way to reconciliation of models that specify impulsivity as a reflective indicator of externalization with those treating it as an indicator of internalization (see Markon et al., 2005). This information would inform future research at the interface of personality and substance use etiology by suggesting that when one personality dimension is held constant, a less biased estimate of the other dimension’s effects on substance use might be obtained. In this study, we test our functional model using structural equations and undergraduate student data.

## MATERIALS AND METHODS

### PARTICIPANTS AND PROCEDURES

This study recruited undergraduate students (*n* = 270) from the Towson University Psychology Research Pool. Participants were 33.5% male (*n* = 91), 19.41 years old on average, and ranged from 18 to 25 years in age. In addition, 82.3% of participants identified as White, 12% Black, 1.9% Asian or Pacific Islander, 2.3% Hispanic, and 1.5% other. All undergraduate students enrolled in general psychology and other psychology-related courses could sign up for the study through the Research Pool website. Students electing to participate in the study chose the date and time to complete surveys from a list of available times. Upon entering the testing room, participants received a consent form, reviewed orally by the experimenter, outlining the general purpose of the study, possible risks of participation, contact information for the study investigators, and participant compensation. Participants received two research pool credits to be used to fulfill course requirements or gain extra credit.

### MEASURES

We selected subscales from several well-known personality questionnaires for use as indicators of latent variables representing reward and threat sensitivities. Because of the large number of items in the subscales, we decided to observe them for adequate internal consistency (i.e., coefficient alpha; Cronbach, 1951) and then compute item composites to avoid a problematic case to parameter ratio (i.e., low statistical power). This procedure produced a case to parameter ratio of ∼5–1, satisfying the minimum often indicated for structural equations modeling (SEM) studies (Bentler and Chou, 1987). Therefore, we expected that the study would have adequate power. Below we describe the scales used to measure our constructs (see also **Table 1**).

**Table 1:** Correlation Matrix.

	ExSeek	TAS	NEOexc	NEOself	NEOvuln	NEOanx	stai	NEOimp	Lifetime	Recent
ExSeek	1.950									
TAS	0.471**	2.186								
NEOexc	0.426**	0.531**	4.729							
NEOself	-0.028	-0.061	-0.072	4.693						
NEOvuln	-0.086	-0.065	-0.118	0.555**	4.715					
NEOanx	-0.098	-0.128*	-0.157**	0.577**	0.646**	4.728				
stai	-0.013	-0.057	-0.157**	0.578**	0.737**	0.661**	9.385			
NEOimp	0.073	0.061	0.094	0.357**	0.412**	0.432**	0.469**	4.335		
Lifetime	0.284**	0.248**	0.210**	0.030	0.075	-0.002	0.120*	0.164**	1.651	
Recent	0.196**	0.179**	0.255**	0.006	0.068	-0.066	0.051	0.244**	0.632**	3.371

Standard deviations displayed along the diagonal. *p < 0.05, **p < 0.01.

#### Reward sensitivity

In this study, we measured latent reward sensitivity using the thrill and adventure and experience seeking (ES) subscales of the Brief Sensation Seeking Scale (BSSS), along with the excitement seeking subscale of the NEO-PI. Although we discussed the possibility that affects such as happiness and sadness might reflect reward and threat sensitivities, we did not include indicators of these affects because reward and threat sensitivities may not produce them directly. According to Carver (2008), these affects may emerge from a feedback process that tracks how well the approach and inhibition systems are doing. Robinson and Berridge (2008) also indicated that wanting and liking can be distinguished at the neural level and are sometimes experienced independently. Thus, happiness may be relatively distinct from eagerness and excitement. Similar, sadness may be relatively distinct from anxiety.

The BSSS is characterized by five point Likert scales and does not reference specific risk behaviors. In a sample of 6,368 adolescents, high BSSS scores were linked to positive attitudes toward drugs and both lifetime and past 30 day use of alcohol, cannabis, inhalants, psychedelics, and cocaine/crack (Hoyle et al., 2002). High BSSS scores also predicted intention to try cannabis and intention to use cannabis frequently. The complete BSSS scale contains eight items and four subscales, including DIS, thrill and adventure seeking (TAS), boredom susceptibility (BS), and ES. We used the TAS and ES subscales in this study because we theorized that they directly reflected the tendency to seek out and experience extreme levels of positive emotion. We excluded BS and DIS because while they are clearly correlates of reward sensitivity, they may reflect this construct only indirectly or function differentially as its indicators. For example, DIS has reflected NEO impulsivity in prior research (Whiteside and Lynam, 2001). Given that the TAS and ES are comprised of only two items each, we reasoned that coefficient alpha would underestimate subscale reliability. Therefore, we conducted a confirmatory factor analysis to examine whether each scale’s items reflected their respective construct. We tested a two factor model with 1° of freedom and fit to the data was excellent (i.e., χ^2^ = 0.008, *p* = 0.93; CFI = 0.99; TLI = 1.00; and RMSEA = 0). Standardized factor loadings ranged from β = 0.50 to 0.75, suggesting the items provided valid measurement of their constructs.

As mentioned above, we also used the excitement seeking subscale of the NEO-PI (NEO-ES) to construct latent reward sensitivity. The NEO-PI is a widely used personality inventory with considerable empirical data to support its internal and external validity (Costa and McCrae, 1992). The NEO-PI subscales consist of eight statements describing particular behaviors. Participants rate the degree to which each statement is applicable to them on a five point Likert scale (i.e., from “strongly disagree” to “strongly agree”). Researchers have reported adequate levels of internal consistency for NEO-ES (i.e., α = 0.89; Eggert et al., 2007). For this study, coefficient alpha for NEO-ES was observed at α = 0.65.

#### Threat sensitivity

In this study, we constructed latent threat sensitivity using NEO-PI anxiety (NEOanx), self-consciousness (NEOselfconsc), vulnerability to stress (NEOvulntostr), and the Anxiety Sensitivity Index (ASI).

Researchers have reported high levels of internal consistency for the neuroticism facet of the NEO-PI (i.e., α = 0.92; Eggert et al., 2007). Research also indicates that the NEO-PI demonstrates strong test-retest reliability for neuroticism over 6 years (*r* = 0.83; Costa and McCrae, 1992). For this study, coefficient alphas for the NEO-PI neuroticism subscales, including NEOanx, NEOselfconsc, and NEOvulntostr, were observed at α = 0.70, 0.68, and 0.77.

We used the original version of the ASI (Reiss et al., 1986) in this study because we believed it was most applicable to our participants. Although a revised version of the ASI has been developed in order to improve content validity and internal consistency (ASI-R; Taylor and Cox, 1998), Deacon et al. (2003) found that the content of the ASI-R was sometimes inapplicable to the experiences of college students. The ASI has demonstrated adequate psychometric properties. In a study of 420 undergraduate students, researchers found that the ASI demonstrated adequate levels of internal consistency (α = 0.74) and concurrent validity. In particular, the ASI and negative affect were correlated at *r*= 0.35, while the ASI was not significantly correlated with positive affectivity (Vujanovic et al., 2007). For this study, coefficient alpha for the ASI was observed at α = 0.88.

#### Impulsivity

This study used NEO-impulsiveness (NEOimp) to measure impulsivity. NEOimp measures the tendency to act on cravings and urges rather than controlling them and delaying gratification. As mentioned, the NEO-PI is a widely used personality inventory with considerable empirical data to support its internal and external validity (Costa and McCrae, 1992). Prior research has reported the internal consistency of the impulsiveness subscale at α = 0.63 (Whiteside and Lynam, 2001). For this study, coefficient alpha was observed at α = 0.63.

#### Substance use

We measured substance use with the Alcohol, Smoking, and Substance Involvement Screening Test (ASSIST; WHO ASSIST Working Group, 2002). The ASSIST consists of eight questions that measure lifetime substance use, recent use frequency, use-related consequences, risk of harm, dependence, and intravenous drug use. Each question addresses nine substance categories: tobacco, alcohol, cannabis, cocaine, amphetamine, inhalants, sedatives, hallucinogens, opioids, and other. In a study of 1,047 participants aged 18–45, internal consistency for the ASSIST was observed at α >0.80 for most domains (Humeniuk, 2006). We used the lifetime drug use and drug use during the past 3 months subscales to construct latent substance use. Prior research has demonstrated the validity of both of these subscales. For example, the lifetime drug use subscale has been positively correlated with lifetime use as measured by the Mini International Neuropsychiatric Interview (*r* = 0.93), and the recent use subscale has been positively correlated with recent frequency of use as measured by the Addiction Severity Index (*r* = 0.84; Humeniuk, 2006). For this study, we computed Guttman’s λ for the lifetime items because they were binary and observed it at λ = 0.71. The internal consistency of the past 3 months drug use subscale was observed at α = 0.63.

### ANALYSIS

Prior to our analyses, we looked for violations of the assumptions SEM. No evidence of outliers was found (i.e., leverage values 5 times greater than the sample average) and no skew statistics larger than 2 were observed. The percentage of data missing for the variables in this study ranged from 0 to 2.9%. Given that the prevalence of missing data was trivial, we used the expectation maximization (EM) algorithm available in the SPSS 22 Missing Values Package to produce a stochastic imputation (for a discussion of when stochastic imputation is appropriate, see Enders, 2010, p. 50). Using the EM algorithm to impute missing data provided us with a single set that allowed us to compute scale scores in a very straightforward manner. In addition, this procedure allowed us to avoid the use of average fit indices that are provided in the context of multiply imputed sets. Stochastic regression-based imputation procedures produce unbiased parameter estimates under the assumption of missing at random (MAR; Enders, 2010, p. 48). We observed that Little’s MCAR test yielded a non-significant result (χ^2^ = 191.68, *df* = 180, *p* = 0.26; Little and Rubin, 2002), suggesting that the MAR assumption was satisfied and the data were likely missing completely at random.

In this study, we used (SEM) and the one step approach advocated by Leslie Hayduk and colleagues to test our hypothesized structural model (see Hayduk and Glaser, 2000; Hayduk et al., 2007). We carried out our analyses in MPlus 6.11, used raw data as input, used Maximum Likelihood as the estimator, and conducted all tests of statistical significance at the *p* < 0.05 level.

#### Hypothesized structural model

Our hypothesized structural model included latent reward and threat sensitivities as exogenous variables and NEO impulsivity and substance use as endogenous variables. We hypothesized that our exogenous variables impacted substance use indirectly through impulsivity. Evidence of these relations would suggest that impulsivity might mediate the effects of reward and threat sensitivities on substance use. In addition, our review of the literature indicated that impulsivity may stem from difficulty shifting attention between reward and threats, which might be exacerbated by greater sensitivity to stimuli of both valences. Thus, we planned to test the possibility that reward and threat sensitivity interact to predict impulsivity, which in turn predicts substance use. We also planned to specify an effect between the latent reward and threat sensitivity interaction and substance use to examine whether impulsivity seemed to mediate the interaction’s effect on substance use.

#### Goodness of fit criteria

This study used a variety of fit indices because they provide different information about model fit. We considered the substantive meaningfulness of the model, significant χ^2^ statistics as evidence that models did not fit the data exactly (Bollen, 1989; Kline, 2010), Tucker-Lewis (TLI) and comparative fit (CFI) indices >0.95 evidence of good fit (Hu and Bentler, 1999; Byrne, 2001), and root means square error of approximation values of <0.05 to be acceptable (RMSEA; Browne and Cudeck, 1993).

## RESULTS

### STRUCTURAL EQUATIONS MODELING

**FIGURE 1:**
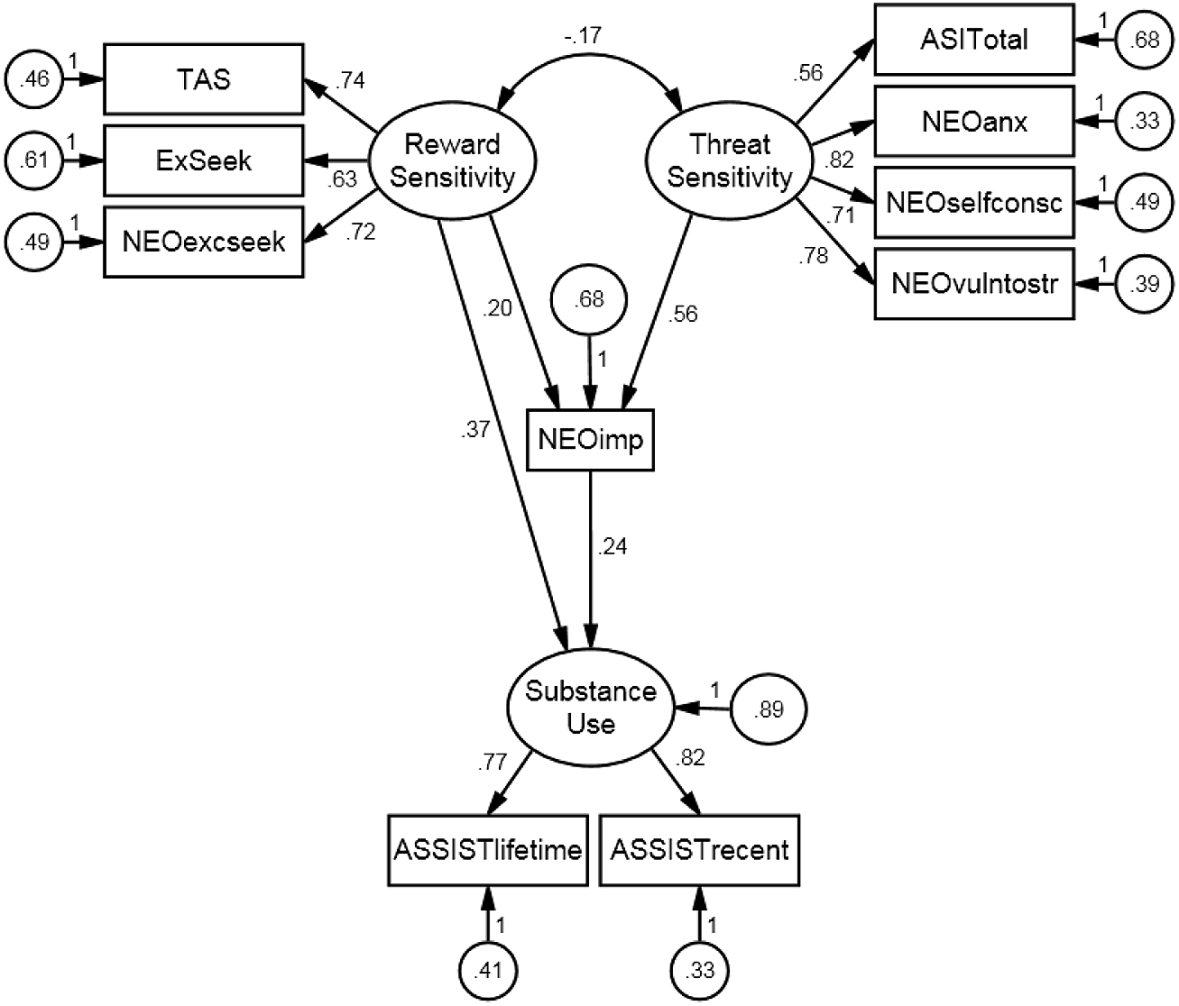
**Final SEM – statistically significant standardized estimates displayed**.

We tested our hypothesized structural model (see **Figure 1**), observed that it was over-identified with 30° of freedom, and fit to the data was excellent (χ^2^ = 36.21, *p* = 0.20; CFI = 0.99; TLI = 0.99; and RMSEA = 0.03). Because the χ^2^ was not statistically significant, we did not reject the hypothesis that the model fit the data exactly (Bollen, 1989, pp. 263–269; Kline, 2010). In addition to interpreting fit information, we interpreted the substance of the model’s statistically significant parameter estimates (see **Table 2**). First, we observed the standardized effects of latent variables on their reflective indicators. We observed that reward sensitivity had large effects on its indicators, including TAS (β = 0.74), BSSS experience seeking (β = 0.63), and NEO-PI excitement seeking (β = 0.72). We also observed threat sensitivity had large effects on NEOanx (β = 0.82), NEOselfconsc (β = 0.71), NEOvulntostr (β = 0.78), and the ASI (β = 0.56). Finally, latent substance use had large effects on life-time (β = 0.77) and recent substance use (β = 0.82). These large effects suggested that our scale scores provided valid measurement of reward sensitivity, threat sensitivity, and substance use.

**Table 2:** Unstandardized and standardized parameter estimates.

			*B*	SE	*p*	β
ExSeek	←	Reward sensitivity	1.000	–	–	0.626
TAS	←	Reward sensitivity	1.316	0.164	< 0.001	0.735
NEOexcseek	←	Reward sensitivity	2.770	0.362	< 0.001	0.715
NEOanx	←	Threat sensitivity	1.000	–	–	0.819
NEOselfcon	←	Threat sensitivity	0.865	0.076	< 0.001	0.713
NEOvulntostr	←	Threat sensitivity	0.948	0.075	< 0.001	0.779
ASITotal	←	Threat sensitivity	1.502	0.171	< 0.001	0.563
ASSISTlifetime	←	Substance use	1.000	–	–	0.770
ASSISTrecent	←	Substance use	2.178	0.379	< 0.001	0.822
Reward sensitivity	→	Substance use	0.380	0.108	< 0.001	0.365
Threat sensitivity	→	Substance use	-0.012	0.031	0.692	-0.038
NEOimp	→	Substance use	0.071	0.025	0.004	0.242
Reward sensitivity	→	NEOimp	0.723	0.234	0.002	0.204
Threat sensitivity	→	NEOimp	0.627	0.072	< 0.001	0.560
Reward sensitivity	↔	Threat sensitivity	-0.801	0.377	0.034	-0.170

Next, we observed between construct correlations and standardized regression coefficients. As hypothesized, there was a small negative correlation between reward and threat sensitivities (*r* = -0.17). We examined whether our model induced this correlation by conditioning on colliders (e.g., latent substance use; see Pearl, 2009, p. 336) and found that this did not appear to be the case. When only reward and threat sensitivities were modeled, their correlation was also *r* = -0.17. Colliders are endogenous variables characterized by two or more incoming causal paths. In our model, the effects of reward and threat sensitivity collide in impulsivity and also substance use. Colliders are salient because conditioning on them can induce artificial correlations between variables that cause them. To illustrate, if we are modeling “contracted a disease or not” as an endogenous variable caused by two independent factors, a negative correlation may be induced between the causal factors because once we know someone contracted the disease and was characterized by the first factor, it becomes correspondingly less likely that they also had the second factor.

Moving on to standardized regression coefficients, reward sensitivity had a small positive direct effect on impulsivity (β = 0.20), while threat sensitivity had a large positive direct effect on this construct (β = 0.56). Impulsivity appeared to mediate the effect between threat sensitivity and substance use completely, while partially mediating the effect between reward sensitivity and substance use. Reward sensitivity had a moderate direct effect on substance use (β = 0.37) and a small but significant indirect effect on this construct (β = 0.05, *p* < 0.05). Threat sensitivity did not have a significant direct effect on substance use, but had a small and significant indirect effect on this construct (β = 0.14, *p* < 0.05). These results suggest that within levels of impulsivity, reward sensitivity had a significant positive effect on substance use, while threat sensitivity did not.

Finally, we used numerical integration to estimate the effects of a latent interaction between reward and threat sensitivities on impulsivity and substance use. In MPlus 6.11, fit statistics are not provided in the context of latent interactions. However, Mooijaart and Satorra (2012) recommend that it is good practice to simply make sure fit is good without latent interactions and then add them. Standardized coefficients are also not available in this context because dependent variable variances vary over observations. We found that *b* = 0.006 for the effect of the latent interaction on substance use (*p* = 0.80) and *b* = 0.13 for its effect on impulsivity (*p* = 0.035). The latter suggested that reward and threat sensitivities’ direct effects on impulsivity and indirect effects on substance use were larger when participants were characterized by higher levels on both sensitivities. Finally, the non-significant direct effect of the interaction on substance use suggested that impulsivity fully explained the effect between these variables.

## DISCUSSION

This study used SEM and undergraduate student data to examine the effects of reward and threat sensitivities on substance use, along with the extent to which impulsivity explained these effects. Our results suggest that impulsivity may translate inversely related reward and threat sensitivities into substance use, completely mediate the effect between threat sensitivity and substance use, and partially mediate the effect between reward sensitivity and substance use. Our results also suggest that individuals with a combination of higher levels on both reward and threat sensitivities may be most impulsive and vulnerable to substance use. These findings provide support for Newman and colleagues’ three pathways to impulsivity, including a reward mediated pathway, a threat mediated pathway, and a pathway through disintegrated attention to reward and threats. They are also consistent with the functional model we developed. Finally, our model’s estimates imply that heightened reward sensitivity may drive substance use, holding impulsivity constant; meaning that among people with the same level of impulsivity, greater sensitively to reward predicts heightened substance use.

### IMPLICATIONS FOR RESEARCH

This study suggests some interesting directions for individual differences research. From an evolutionary perspective, levels of reward and threat sensitivity, as well as rash action or impulsivity, are expected to calibrate adaptively to environmental conditions early in development (Del Giudice et al., 2011; Ellis et al., 2012). Bearing on this, research suggests that greater risk-taking, sensation seeking, and executive dysfunction stem from habitation in harsh and unpredictable environments (for a review, see Ellis et al., 2009). The expression of these personality traits may also be sensitive to less obvious cues in the environment, such as sex ratio (Ellis et al., 2012). Thus, our model might be used to link environmental conditions to substance use in future research. This might pave the way to prevention interventions that decrease substance abuse through their effects on personality traits and fundamental dimensions of environment (e.g., unpredictability).

In prior research, inconsistent findings regarding the effect between threat sensitivity and substance use were reported (Staiger et al., 2007). This study suggests that threat sensitivity may have a significant indirect effect on substance use, holding reward sensitivity constant. In future research, conditioning on reward sensitivity could yield more consistent estimates of the effects between threat sensitivity, impulsivity, and substance use. Further, our findings suggest that impulsivity stemmed from reward *and* threat sensitivities. Including impulsivity in future studies of reward and threat sensitivities’ effects on substance use could provide additional testing of whether this construct translates the former into the latter. Given our findings, it also seems reasonable to theorize that impulsivity may reflect externalizing *and* internalizing personality dimensions. Future research can bring more data to bear on this possibility, perhaps leading to a synthesis of models of that specified impulsivity as reflecting either externalization or internalization.

As noted, reward sensitivity had direct and also indirect effects on substance use, while threat sensitivity had only an indirect effect on this outcome. The two indirect effects are consistent with the literature we reviewed including Newman and colleagues’ model and the Verheul (2001) suggestion that harm avoidance may impact substance use indirectly via a pathway through stress responsivity and DIS. Threat sensitivity’s effect on substance use may be explained by urgent threat avoidance behavior coupled with the capacity psychoactive substances have for providing relief. Similar, part of reward sensitivity’s total effect on substance use may be explained by urgent resource seeking and the capacity substances have for providing the experience of resource discovery. The direct effect of reward sensitivity on substance use was not anticipated by our functional model, and suggests that reward sensitivity may drive substance use through an additional pathway. Substances are often used in social, pragmatic, dietary, and other contexts characterized by norms that discourage rash behavior (Durrant and Thakker, 2003). Stemming from this, one possibility is that among similarly impulsive individuals, greater reward sensitivity explains variance in substance use that is occurring in these kinds of contexts, or that is mediated by more deliberative cognition.

This study builds upon the work of Cyders and colleagues by testing causal assumptions about the linkage between tendencies toward reward and threat sensitivities, impulsivity, and substance use. Findings suggested that impulsivity may stem from reward and threat sensitivities and translate these constructs into substance use. Future research can test structural models using longitudinal data to produce stronger causal inferences about these effects (e.g., using panel models or latent growth curve models; see Martens and Haase, 2006). In addition, instrumental variable approaches could be used to produce consistent estimates of the effects of reward and threat sensitivities on impulsivity and/or substance use (see Antonakis et al., 2010). We note that it may be difficult to establish causal relations in the context of Cyders and colleagues’ positive and negative urgency scales. In particular, the items may conflate emotional extremes and impulsivity. For example, using Cyders and colleagues’ items would bar us from carrying out a randomized experiment and using assignment to an intervention condition as an instrument for estimating reward or threat sensitivity’s effect on impulsivity (see Angrist and Krueger, 2001). The urgency items are clearly useful in the context of many research questions. However, when reward and threat sensitivities’ indirect effects through impulsivity are of interest, items tapping one of these constructs at a time may be needed.

### IMPLICATIONS FOR PREVENTION AND TREATMENT

This study’s findings are consistent with many existing prevention and treatment approaches but also provide new information that could be used to identify at-risk individuals, personalize treatments, and possibly identify mechanisms that mediate treatment outcomes. Our model is consistent with the identification of highly threat sensitive individuals as at-risk for substance abuse and the use of clinical interventions to target threat sensitivity. Currently, substance abuse prevention and treatment both intervene on adolescents and young adults by teaching healthy behaviors, establishing self-efficacy and social support, teaching effective coping skills, and other strategies known to improve mental health [i.e., decrease neuroticism or mental illness; Hansen et al., 2007; National Institute on Drug Abuse (NIDA), 2009]. In this context of our model, these interventions can be understood as impacting threat sensitivity and thereby its indirect effect on substance abuse through impulsivity.

Our model is also consistent with targeting self-regulation or executive function to prevent and treat SUDs. Both prevention and treatment interventions have focused on forms of deliberate thinking, including problem solving, planning, and other skills that promote self-regulation (Hansen et al., 2007; National Institute on Drug Abuse (NIDA), 2009). In the context of our model, this focus on self-regulation can be seen as intervening on impulsivity and thereby two of the three pathways to substance use: the indirect effects of reward and threat sensitivity on substances use through impulsivity.

This study’s findings imply that highly reward sensitive individuals are at-risk for substance abuse. Prevention specialists and clinicians may be able to prevent or reduce high levels of use, respectively, through intervention on this trait or by moderating its effects on impulsivity and substance use. Both prevention and treatment programs have frequently attempted to change the expected utility of substance use through interventions such as mass media campaigns (Klesges et al., 2009) and motivational interviewing (Miller and Rollnick, 2004). For example, many media campaigns have displayed messages designed to help the public frame substances as threats instead of resources, while motivational interviewing assists clients in exploring and processing the outcomes associated with substances use, thus facilitating change in their valuation. In the context of our model, these interventions can be understood as moderating the effects of reward sensitivity on substance use.

Reward sensitivity has received less attention as the actual target of intervention. Alcoholics Anonymous and treatment programs recommend that people attempting to recover from addictions avoid romantic partnerships and other sources of extreme excitement. However, to our knowledge these recommendations stem from personal experiences and anecdotal evidence. We have not seen any prevention campaigns aimed at decreasing the levels of fun and excitement people seek out and experience. We also suspect that this intervention target has often been neglected in treatment settings. Given these gaps, we recommend more research focusing on strategies that may reduce reward sensitivity.

Finally, our findings imply that individuals characterized by a combination of high levels of reward and threat sensitivity should receive special attention as potential targets of prevention and treatment interventions. Clinicians and prevention specialists may reduce or prevent substance abuse among these persons by focusing intervention on all of the variables discussed above, including reward sensitivity, threat sensitivity, and impulsivity (or executive functions and self-regulation). Such interventions could also focus on moderating the significant effects in our model as discussed previously.

Above we have briefly illustrated how prevention and treatment could be integrated according to the areas of our model that they target. Perhaps our model provides a useful conceptual tool for thinking about the concurrent provision of prevention and treatment. Such a tool might be especially useful to colleges and universities that are interested in preventing substance-related harms given that college students are often in need of both prevention and treatment. Further, adolescents and young adults are generally more externalizing, reward sensitive, or have more sensational interests than children or middle adults (Figueredo et al., 2006). Thus, academic institutions may yield significant benefits from prevention and treatment interventions that attend to reward sensitivity in addition to threat sensitivity and self-regulation.

### LIMITATIONS

Several limitations apply to inference based on this study’s results. This study is limited by the undergraduate sample, the use of self-report data, and the absence of temporal information. Inferences drawn from these data should not be extrapolated to populations that are differentially aged and should be cautiously applied to young adults inhabiting different environments. The limitations of self-report data are now well-known. In spite of their limitations, self-report data are economical and robust enough that their use is very common. These data were cross-sectional and thus causal inference must remain tentative. In spite of its limitations, this study produced valuable new information that can inform research at the interface of personality and substance use; build upon recent work that has addressed the nature of impulsivity; and drive enhancement of prevention and treatment programs, including those carried out by colleges and universities.

## Conflict of Interest Statement

The authors declare that the research was conducted in the absence of any commercial or financial relationships that could be construed as a potential conflict of interest.
